# IMPACT OF END-OF-LIFE PLANNING ON SURROGATE DECISION-MAKER ANXIETY

**DOI:** 10.1093/geroni/igz038.406

**Published:** 2019-11-08

**Authors:** April Aloiau, Daniel L Segal, Alan Mouchawar, Melissa J Benton

**Affiliations:** 1 University of Colorado at Colorado Springs, Colorado Springs, Colorado, United States; 2 Hoag Hospital, Newport Beach, California, United States

## Abstract

At the end of life, adults with advanced illness frequently rely on surrogate decision makers to make health care decisions. Surrogate decision makers often have anxiety related to the difficulty and complexity of making end of life decisions. This project evaluated whether an educational intervention focused on creating a specific plan of care for hospice patients would reduce anxiety among their surrogate decision makers. The Geriatric Anxiety Scale (GAS), the State Trait Anxiety Inventory–State Anxiety Scale (STAI-S), and a single question about decision-making anxiety were used to measure surrogate decision maker anxiety before the intervention, immediately after the intervention, and 2 weeks following the intervention. After completing an informed consent, 12 patients (age 80 ± 14.7 years) and 18 surrogate decision makers (age 60 ± 12.9 years) from a Southern California hospice organization participated in the educational intervention. Immediately following the intervention surrogate decision maker anxiety decreased. Mean GAS anxiety scores decreased (p = 0.003) from 21.3 ± 9.8 to 16.6 ± 7.6 and STAI-S scores decreased (p = 0.003) from 43.3 ± 11.5 to 38.1 ± 9.9. However, when surrogate decision maker anxiety was measured 2 weeks post-intervention, anxiety had increased again, so that it was no longer significantly different from pre-intervention levels. Qualitative analyses showed high satisfaction, with 85% of decision makers reporting that the education was very or extremely helpful This project demonstrated that an educational intervention in the hospice setting can be effective in creating a short-term decrease in surrogate decision maker anxiety levels.

